# Effect of Fillers Modification with ILs on Fillers Textural Properties: Thermal Properties of SBR Composites

**DOI:** 10.3390/ijms25020885

**Published:** 2024-01-10

**Authors:** Magdalena Gaca, Cyril Vaulot

**Affiliations:** 1 Department of Chemistry, Institute of Polymer and Dye Technology, Lodz University of Technology, 16 Stefanowskiego Street, 90-537 Lodz, Poland; 2 Institut de Science des Matériaux de Mulhouse (IS2M), Université de Haute-Alsace, CNRS UMR 7361, 15 rue Jean Starcky-BP 2488, CEDEX, 68057 Mulhouse, France; cyril.vaulot@uha.fr

**Keywords:** nanocomposites, graphene nanoplatelets, SBR, textural properties, thermal properties

## Abstract

In this work, we present the effect of graphene nanoplatelets (GnPs) modification with ionic liquids (ILs). The textural properties of graphene nanoplatelets (GnPs) used as styrene-butadiene rubber’s filler and the thermal properties of the composites obtained with the use of the mentioned fillers were investigated. GnPs were modified with 1-butylpyridinium bromide (BPyBr) and 4-methyl-1-butylpyridinium bromide (BmPyBr) through two different ways. One strategy has been to deposit the filler modifier from the solution. The second one involved the modification of the filler with ionic liquids in bulk during the preparation of elastomer blends. Settlement of the proposed ionic liquids onto the GnPs’ surface led to significant changes in the textural characteristics. BPyBr has restricted the filler’s microporosity, whereas BmPyBr has caused the formation of a more opened filler structure without the increase in its average pore size. GnPs modified with ILs led to reducing the temperature of vulcanization of SBR compounds and affected the thermal stability of the composites.

## 1. Introduction

Polymer composites occupy an undisputed place in polymer technology. The importance and applicability of these materials increase when fillers are used to prepare them.

Among the many fillers of polymer composites, it is the carbon ones that have been successfully used in rubber processing for several decades. Carbon black is primarily a filler commonly used to improve the properties of rubber products. Among scientists, carbon materials with a structure different from the popular carbon black are also of great interest. This includes, e.g., carbon nanotubes, graphite and graphene [1,2,3,4,5,6,7].

Graphene has been repeatedly described as a carbon material with unique properties that make it applicable, for example, in sensors, electronics and composites [8].

Despite its unique properties, the use of graphene in polymer processing is still limited. It is important to homogeneously disperse graphene in the polymer. Using various methods, attempts were made to improve the dispersion of graphene fillers in polymers [9]. It is crucial to reduce the trend towards graphene particle aggregation and restacking due to strong inter-sheet forces in order to decrease their surface free energy.

For many years, scientists have studied ionic liquids (ILs) that can be used effectively as a medium for the chemical reactions or compounds in materials preparation. These substances are liquid salts at room temperature, with melting points less than 100 °C. They are considered as environmentally friendly due to their almost zero vapor pressure, relative non-flammability, high heat capacity and thermal stability [8].

In our previous work, we studied the possibility of modifying GnPs with the use of two ILs through two different strategies [10]. The ILs were introduced into rubber as a single component (melt-mixing method) or via immobilization onto GnPs from solution. We described the detailed procedure of GnPs modification with the use of 1-butylpyridinium bromide and 4-methyl-1-butylpyridinium bromide. The methodology of rubber mixes and composites preparations was featured carefully (which will enable other researchers to replicate this strategy) as well as their properties (e.g., rheometrical, mechanical). The effectiveness of the GnPs surface modification process with ionic liquids from the solution was confirmed by the FTIR spectra of the discussed fillers. The amount of ILs settled onto the GnPs surface was estimated by TGA as well. It appeared that GnPs modified in these ways influenced the curing behavior of rubber compounds (optimal time of curing and scorch time). The immobilization of IL with a methyl pendant group on the GnPs surface yielded the composite with the highest modulus and tensile strength. SEM micrographs of studied SBR composites revealed better dispersion of GnPs/IL in the rubber matrix. It was shown that the modified fillers efficiently influenced the photo-actuation ability of the composites filled. Additionally, such high photo-actuation constraint has not been reported in cross-linked systems (in similar conditions) earlier. Thus, the obtained results may be of interest to scientists involved in designing and obtaining multifunctional rubber composites.

Considering the exceptional properties of the elastomers with GnPs modified with ILs, it seemed to be interesting to supplement the previous characteristics of both the fillers and SBR composites.

In this paper, first, the textural properties of GnPs modified with the use of ionic liquids were studied by the N_2_ adsorption method. We have successfully moderated the textural properties (e.g., surface area) of fillers used by applying two different ionic liquids, namely 1-butylpyridinium bromide (BPyBr) and 4-methyl-1-butylpyridinium bromide (BmPyBr). Then, these additives were applied in an SBR matrix to investigate the effect of surface modification on the thermal behavior of SBR nanocomposites.

## 2. Results and Discussion

### 2.1. Textural Properties of Pristine GnPs and GnPs Modified from Solution with 1-butylpyridinium Bromide (BPyBr) and 4-methyl-1-butylpyridinium Bromide (BmPyBr)

**Figure 1:**
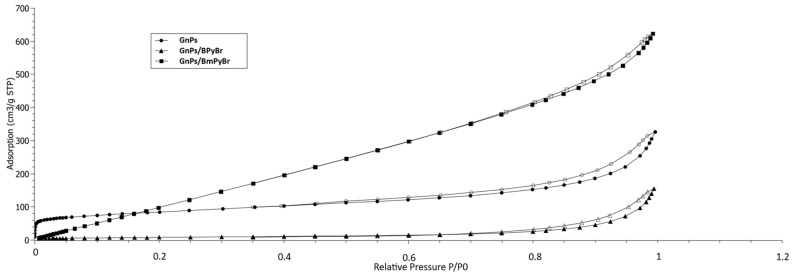
Volume of adsorbed gas dependence on relative pressure of pristine and modified GnPs.

The textural properties of three nanoporous fillers have been evaluated using gas adsorption at cryogenic temperatures. The nitrogen adsorption/desorption isotherms of pristine and modified-from-solution GnPs provide information about these properties (here from a point of view of potential filler—polymer interactions). Isotherms are shown for the three samples evaluated (Figure 1). Some differences in this have been revealed. Untreated GnPs show nitrogen adsorption typical for microporous materials [11]. Adsorption and desorption isotherms at high pressure are not identical. The hysteresis phenomena indicated the capillary condensation and the presence of mesopores (2 ≤ *Θ* ≤ 50 µm). The shape of the hysteresis loop (around 0.45 *P/P*_0_) corresponds to H4-hysteresis, describing the adsorption between the layers of microporous material. After modification of GnPs with the use of both ionic liquids, no N_2_ adsorption at relatively low pressure occurred.

The Brunauer–Emmett–Teller model considers the statistical pressure where the surface of the adsorbent is covered by a monolayer of the adsorbate [12]. This covering enabled designation of the specific surface area (*S_BET_*) of all investigated GnPs-based fillers (Table 1). GnPs modification with BPyBr led to a significant reduction in the filler’s *S_BET_*, whereas the filler’s treatment with BmPyBr yielded *S_BET_* 2.5 times more than pristine GnPs. *S_BET_* of the untreated particles was almost 300 m^2^ g^−1^. Due to the treatment of GnPs with BPyBr, the filler’s microporosity was cancelled, leading to a decrease in its total adsorbed volume *V_max_* (reaching only 35 cm^3^ g^−1^ STP). On the other hand, GnPs/BmPyBr was characterized by the highest value of *V_max_* among all samples investigated. Probably, BmPyBr took part in the formation of the filler’s structure with larger pore volumes. Insight into the textural properties of investigated fillers was assessed by the average pore size (*D*). An increase in the average pore size was found for GnPs/BPyBr, while the average pore size of GnPs modified with BmPyBr remained almost unchanged.

**Figure 2:**
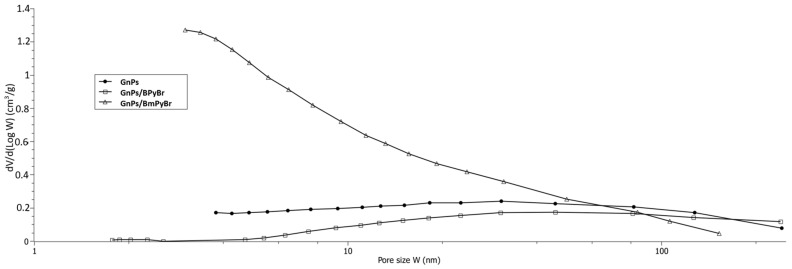
BJH distribution of the pore size of pristine and modified GnPs.

To study the mesoporosity of the samples, the BJH model combining the adsorbed volume for each *P/P*_0_ with a pore size was applied [13]. In that case, pores are treated as cylindrical, opened and not-interconnected individuals. The BJH model does not refer to microporosity. Therefore, the results of the BET and the BJH models are quite different for samples in which microporosity occurs. As GnPs/BPyBr is discussed, the *S_BJH_* value corresponds to the absence of micropores and strengthens the *V_max_* values (Table 1). In the case of pristine GnPs and GnPs/BmPyBr, almost half of the BET surface was studied by the BJH model as roughly one. The BJH model leads to the mesopores distribution (Figure 2).

When GnPs were not modified with ionic liquids, a large distribution of mesoporosity with a maximum of about 24 nm was observed. Settlement of BPyBr onto GnPs surface caused a very large distribution of pore sizes ranging from 50 to 1000 nm. A very large and decreasing distribution of the mesoporosity with small pores between 5 and 150 nm became visible when the filler was treated with BmPyBr.

Insight into the microporosity was assessed by the Dubinin–Astakhov model [14]. Since no micropores for GnPs/BPyBr have been noticed, this model should not be applicable (Table 1). It appeared that the treatment of GnPs with BmPyBr led to the development of a microporous surface and volume of the filler.

**Figure 3:**
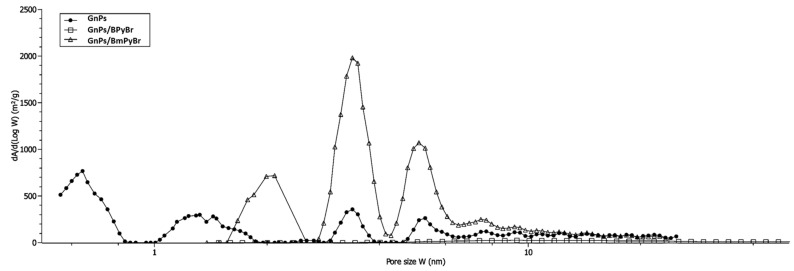
DFT pore size distribution for pristine and modified GnPs.

Using the DFT model [15], a global pore size distribution has been given (Figure 3).

Pristine GnPs were characterized by a multimodal distribution of the porosity with three main and narrow pores’ diameters (0.65, 1.3 and 3.4 nm). This simulation led to knowing more completely the effect of the treatment. When GnPs/BPyBr had no micropores, a large distribution with some large pores was observed. As a result of the filler’s surface modification with BmPyBr, some parts of very small pores have been lost. This was proved by the DFT showing bimodal and narrow pore size distribution (Figure 3).

### 2.2. Curing Kinetics of SBR Compounds by DSC Measurements

**Figure 4:**
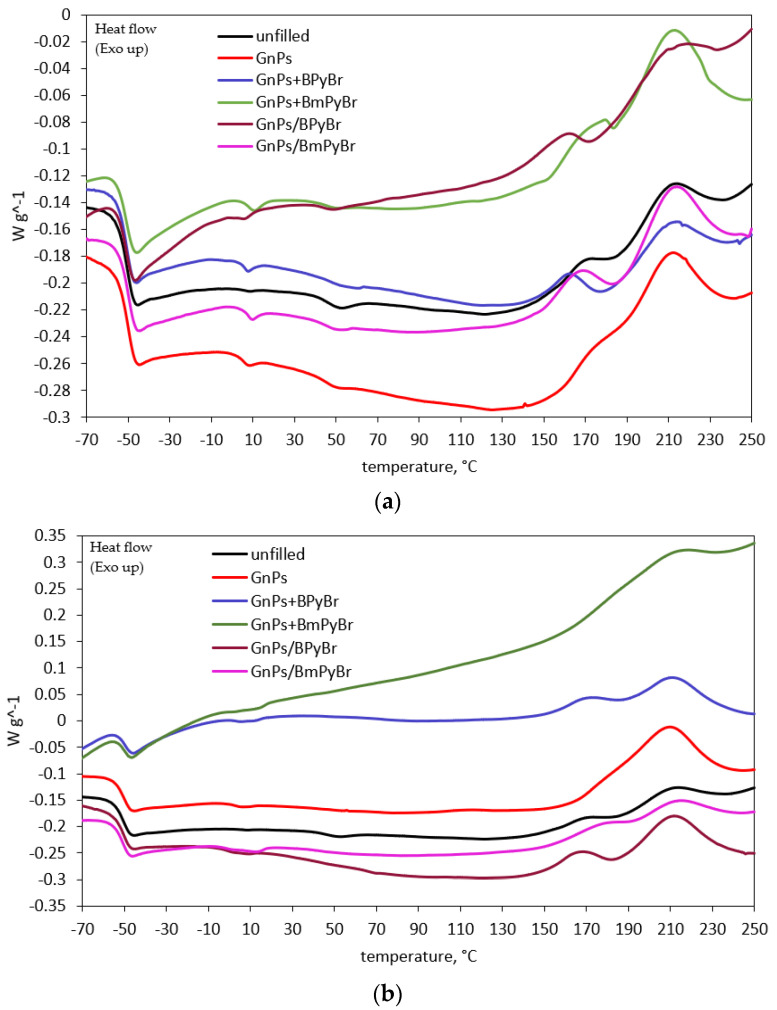
DSC curves of SBR unfilled compound and compounds filled with: (**a**) 1 phr of GnPs and (**b**) 5 phr of filler.

Using differential scanning calorimetry, a single glass transition at the temperature of about −50 °C was observed for composites containing 1 or 5 phr of GnPs (modified with ILs from solution or in bulk). An unfilled sample was studied as well (Figure 4).

The glass temperature (*T_g_*) and heat capacity (Δ*c_p_*) of the polymer at glass transition are quoted in Table 2. The applied fillers slightly affected the *T_g_*, leading to no systematic dependencies. Such insignificant differences in the *T_g_* values between composites studied indicate that the average relaxation of bulk segments has remained unchanged by incorporation of the proposed fillers. The change in heat capacity is strongly affected by polymer molecular behavior at glass transition [16] and represents the change in polymer chain mobility in the composite [17]. It can be seen that Δ*c_p_* of unfilled composite is higher than that of SBR GnPs-based samples. Due to the lack of filler particles, rubber macromolecules are unrestricted, thus making a greater contribution to glass transition. It stays in line with mechanical properties, especially sample elongation at break described for these composites in our previous paper [10], since the higher the Δ*c_p_* value, the lower the stiffness of the composite. For all filled and herein-discussed samples, Δ*c_p_* decreased with increasing filler loading, with the exception of samples containing GnPs/BPyBr. The decrement of Δ*c_p_* was more visible when GnPs were modified with ILs in bulk. As Δ*c_p_* is a measure of the amount of the polymer that participates in the glass transition [18], the fraction of immobilized polymer around the filler particles (*χ*) can be calculated [19]. The data are presented in Table 2. The former Δ*c_p_* values are in line with the weight fraction of immobilized rubber. The higher the values of *χ*, the stronger the interfacial interactions between polymer and filler. These observations are consistent with our earlier studies [10]. Evidently, the *χ* values are much higher for samples filled with 5 phr of GnPs modified in bulk with both ILs (BPyBr or BmPyBr).

The impact of the procedure of IL introduction, as well as the kind of IL, was also validated through the temperature range and energetic effects of vulcanization using DSC analysis. This technique is commonly used to study the curing kinetics of polymers [20,21]. The obtained results are given in Figure 4. According to the literature, generally, SBR vulcanization is a two-step exothermic process [22]. The first step proceeds in the lower temperature range, due to the formation of sulfur cross-links between rubber macromolecules, while the second one occurs at higher temperatures.

**Figure 5:**
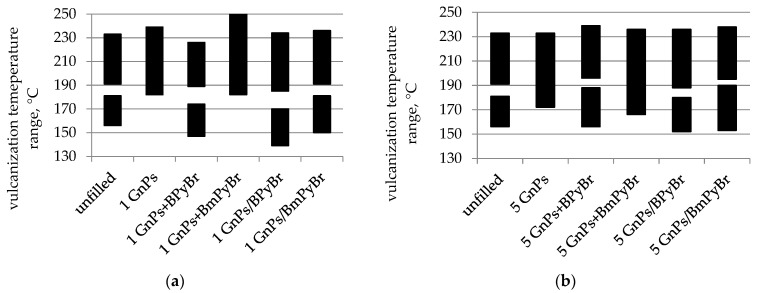
The vulcanization temperature range (*T_C_*) of SBR compounds unfilled and filled with (**a**) 1 phr of GnPs and (**b**) 5 phr of filler.

Then, polymer chain cross-linking is accompanied by other reactions, such as: cyclization, rearrangement and chain fragmentation, shortening of sulfide bridges or the formation of low quantities of volatile products due to their thermal decomposition [23]. However, for the herein-discussed samples with pristine GnPs or GnPs modified in bulk with BmPyBr, a one-step vulcanization process is observed. Then, the curing and “post–curing” reactions occur together. It can be seen that unfilled SBR compound vulcanizates at a temperature range of 156–233 °C with an energetic effect of 5.96 J g^–1^. The addition of the pristine GnPs into SBR compounds led to significant differences in the vulcanization temperature range (*T_C_*) and enthalpy of the vulcanization (Δ*H*) of those samples (Figure 5). Fillers are known to have a catalytic effect on the cross-linking of polymers, leading to the lowering of the vulcanization onset temperature [24]. Nevertheless, the presence of pristine GnPs in studied samples increased the vulcanization onset temperature by 16 °C, when filler loading was 5 phr, or 26 °C, when GnPs loading was 1 phr. However, higher Δ*H* of rubber compounds with neat GnPs in comparison with unfilled samples was observed. The energetic effect increased, reaching the values of 12.7 J g^–1^ and 24.6 J g^–1^ for 1 GnPs and 5 GnPs samples, respectively.

Our earlier studies proved that neat GnPs formed agglomerates within SBR, and the freely dispersed ILs did not enhance the filler dispersion [10]. Thus, the cross-link reactions occurred at higher temperatures. Additionally, filled rubber compounds’ viscosity (confirmed in an earlier paper [10]), higher than for reference samples, hindered diffusion of heat and components of the cross-linking system through the SBR matrix during vulcanization. Hence, Δ*H* decreased from 12.7 J g^–1^ for compounds with 1 phr of neat GnPs to 7.2 and 6.5 J g^–1^ from samples containing 1 phr of filler and BPyBr and BmPyBr, respectively. When the concentration of GnPs in the rubber compounds was 5 phr, a decrease in Δ*H* from 24.6 J g^–1^ to 9.9 J g^–1^ and 16.3 J g^–1^ was observed. The higher the Δ*H*, the more intensive the cross-linking process. It is possible to attribute Δ*H* decrement to the sulfur and accelerator absorption onto the filler’s surface during compounding, thereby reducing the abruptness of SBR cross-linking. The possibility of reducing the temperature of vulcanization of rubber compounds is extremely important from the point of view of polymer processing, i.e., reducing the energy consumption needed to prepare polymer composites. When GnPs and BPyBr were incorporated separately into SBR mixtures, a crucial decrease in onset vulcanization temperature was noted, when compared to filled samples without any IL. The replacement of BPyBr by BmPyBr (within the same strategy of modification) resulted in an increase in the vulcanization onset temperature and the extension of the total vulcanization period to a higher temperature. For samples filled with GnPs and BmPyBr alone, the vulcanization onset temperature decreased with increasing filler concentration from 182 °C to 166 °C, although when compared to samples containing only GnPs, these changes were not significant. In the case of the rubber compounds with GnPs/ILs, regardless of the filler’s loading, lowering of the onset of the vulcanization process was noted. It seems that the improvement of filler’s dispersion in SBR (described in [10]) dominates over the influence of the viscosity of the discussed rubber compounds on their cross-linking process. The filler’s surface area can significantly affect the temperature and enthalpy of rubber cross-linking [25]. It is worth noticing that fillers with ILs deposited on their surface affected the vulcanization process more significantly through the spectacular reduction in the onset of *T_C_*, without major changes in the endset *T_C_*, when compared with samples containing pristine GnPs. GnPs/BPyBr with a surface area yielding 30 m^2^ g^−1^ to a greater extent than GnPs/BmPyBr (with S.A = 767 m^2^ g^−1^) promoted the onset of the vulcanization process. Such a decrease in the vulcanization onset temperature allows SBR to be cross-linked at temperatures lower than 160 °C. Probably, the curing system was less effective in the company of a filler having a high surface area, and then, lower heat release accompanied by SBR cross-linking was observed. GnPs decorated with BmPyBr caused the decrease in Δ*H* in comparison to BPyBr-modified GnPs from the solution.

### 2.3. Thermal Stability of SBR Composites and ILs Alone Used to Prepare Composites

Several papers have indicated that the addition of GnPs into a polymer matrix can significantly improve the thermal stability of the obtained material [26]. Herein, it was anticipated that the presence of ILs may lead to different properties. The data related to the temperatures for 2% and 50% mass loss (respectively *T*_02_ and *T*_50_) during decomposition of vulcanizates are shown in Table 3. In the case of SBR composite without any filler, *T*_02_ was 245 °C. After the addition of pristine GnPs, regardless of their loading, *T*_02_ of vulcanizates increased by about 12–14 °C, reaching values of 257–259 °C. Such a noticeable rise in thermal stability was observed due to the physical barrier of the filler within the polymer matrix, resulting in the limitation of oxygen permeation, and the diffusion rate of volatile products was slowed down [27].

The values of *T*_02_ for composites containing modified GnPs showed no systematic change with filler loading. The presence of BPyBr and BmPyBr introduced separately with GnPs into SBR induced an early thermal decomposition of the rubber as compared to samples containing pristine GnPs without ILs (Table 3). It was noticed elsewhere that ILs less stable thermally than the host polymer matrix may initiate the composite’s decomposition at a lower temperature [23]. Thus, ILs deteriorated the thermal stability of SBR vulcanizates. In the case of the thermal stability of composites with modified GnPs, the crucial finding is the thermal stability of ILs used (the TG curves were presented in our previous paper [10]). BmPyBr has a significantly lower *T*_02_ compared to BPyBr, which is proven by its rapid loss of thermal stability (the TG-derived data are listed in Table 4). BmPyBr decomposition is associated with the release of methyl group, which is reflected in the posted curves. The release of the methyl group from BmPyBr did not lead to immediate degradation of the entire IL molecule.

On the contrary, complete BPyBr decomposition was observed at a lower temperature. Consequently, the enhancement in thermal stability of the samples with ILs did not occur. It was found that *T*_02_ values of elastomers with 1 phr GnPs modified with BPyBr through the melt-mixing method increased by 6 °C when compared with the unfilled sample. With further increase in filler concentration, the thermal stability of composites clearly deteriorated, and *T*_02_ was 233 °C when the filler content was 5 phr. This effect can be attributed to poorer filler dispersion inside the SBR matrix. In turn, composites containing GnPs and BmPyBr, introduced separately into the rubber mixture, were more thermally stable as filler concentration was higher and *T*_02_ increased from 235 °C to 253 °C. BmPyBr had a beneficial effect on the filler’s dispersion in elastomer, which was also confirmed by a significant increase in the electrical conductivity of these composites [10]. The incorporation of GnPs modified from solution did not lead to a *T*_02_ increase. On the contrary, a greater susceptibility to thermal decomposition of these samples was noted with the increase in filler loading. It is clear that fillers obtained as a result of ionic liquid deposition on the GnPs surface did not participate in improving the thermal stability of SBR composites. It seems that the presence of ILs surrounding those deposited on the GnPs effectively blocked the “carbon core” from matrix interaction and, in the first place, underwent thermal decomposition. It was stated that the size of filler particles played a role in the improvement of the thermal stability of composites [28]. It stays in line with the obtained results. GnPs modified with BPyBr from solution characterized by lower *S_BET_* were more efficient as heat and mass barrier than GnPs/BmPyBr was. This barrier built by particles of the filler was more effective at high temperatures in slowing down the composites’ degradation. Next, GnPs having a larger surface area, modified from solution with BmPyBr, favors the thermal decomposition of SBR. Such a methyl-group-releasing filler deteriorated the thermal stability of SBR composites as compared to GnPs/BPyBr.

Notably, the temperature with a mass loss ratio reaching 50% sustained nearly on the same level when pristine GnPs were used to prepare composites (*T*_MAX_ = 445 °C), but for other filled SBR elastomers, *T*_MAX_ was 439 and 443 °C, respectively.

### 2.4. Dynamic Mechanical Analysis of SBR Composites

**Figure 6:**
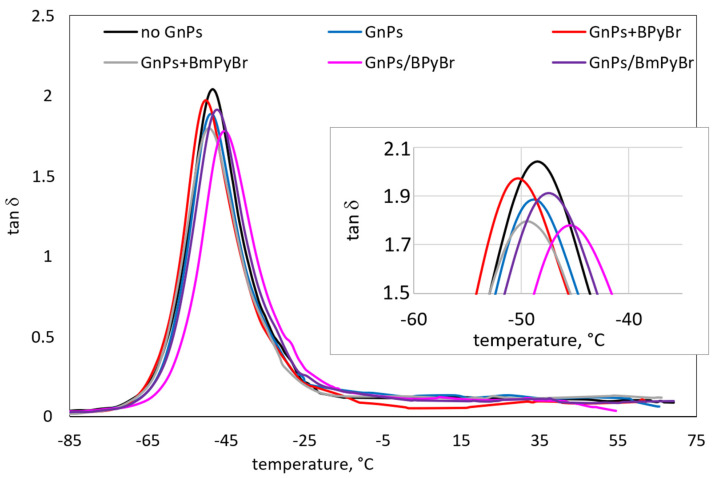
Effect of 1 phr of filler on *tan δ* as a function of temperature for SBR composites.

**Figure 7:**
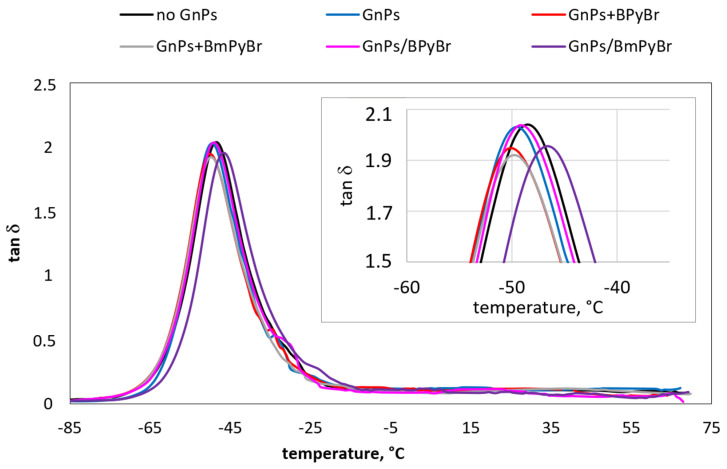
Effect of 5 phr of filler on *tan δ* as a function of temperature for SBR composites.

**Figure 8:**
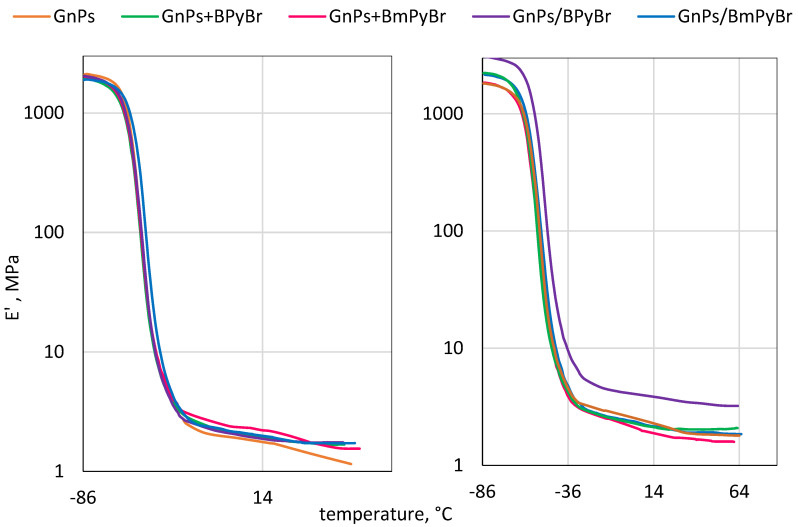
Storage modulus (*E′*) curves versus temperature of SBR composites filled with modified and pristine GnPs: 1 phr of filler (**left**) and 5 phr of filler (**right**).

Dynamic mechanical analysis was used to study the behavior and phase morphology of prepared SBR composites. Therefore, their dynamic mechanical properties are displayed in Figure 6, Figure 7 and Figure 8 and Table 5.

The temperature dependence of *tan δ* for SBR composites filled with different GnPs loading is presented in Figure 6 and Figure 7. The glass temperature (*T_g_*) values of all composites are specified from the peak of loss factor versus temperature dependence curves. The *T_g_* of investigated samples showed a modest increase with the addition of fillers. The increase in filler loading from 1 to 5 phr did not cause any significant differences in the *T_g_* values of the composites filled with pristine GnPs or GnPs modified with ILs in bulk. Obviously, the incorporation of 5 phr of GnPs with BPyBr modified from solution into composite caused shifting the *T_g_* by almost 4 °C to high temperature when compared with composite with a similar amount of filler modified with IL through the melt-mixing strategy. This can be ascribed to the decline in the flexibility of the molecular chain, supported by an increase in the interfacial interactions between the filler and SBR.

The reduction in the *tan δ* peak height (*tan δ_Tg_*) during glass transition can be attributed to restricted segmental mobility of the rubber chains with the addition of GnPs, which resulted in an enhanced SBR−filler interaction. Thus, fewer rubber chains contribute to the loss factor [16]. The *tan δ_Tg_* decreases with filler loading, except for the samples containing GnPs modified in bulk with BPyBr. It is known that the loss factor is a measure of the damping properties of composites, which indicates the ability to convert mechanical energy into heat through internal friction and macromolecular rearrangements [29]. The decrease in *tan δ_Tg_* indicates the enhancement in the dynamic fatigue properties of SBR–GnPs composites. Additionally, the values of loss factor at 0 °C and 60 °C being an assessment of the wet traction and rolling resistance (*tan δ*_0_ and *tan δ*_60_, respectively) are listed in Table 5. A set of low *tan δ*_60_ and high *tan δ*_0_ (contributing to low fuel consumption and good wet grip) is necessary to obtain excellent tire treads [30]. As was discussed elsewhere, wet-skid resistance of the polymer composites could be enhanced by an increase in filler–rubber interactions [31]. Using ILs to modify the low content of GnPs (1 phr) through both proposed strategies led to the deterioration of composites’ skid resistance in comparison with samples containing neat GnPs. On the other hand, *tan δ*_60_ was lower for these composites, indicating enhanced rolling resistance compared to pristine GnPs/SBR composites. Among samples filled with GnPs modified with ILs melt-mixing modification of the filler, regardless of the kind of IL used, enabled us to achieve samples characterized by better skid and worse rolling resistance. At higher concentrations of the modified GnPs, the decrement of *tan δ*_0_ and *tan δ*_60_ was noticed. Taking into account modified GnPs/SBR samples, it was clear that the filler’s modification from solution increased interfacial interactions and led to increased wet traction, but this had no beneficial impact on the rolling resistance of obtained composites.

The curves of storage modulus (*E*′) versus the temperature for studied composites are shown in Figure 8. The storage modulus decreased precipitously with increasing temperature, the result of which corresponds to the glass-rubber transition and enhanced mobility of SBR chains.

For 5 phr of filler, the most visible increase in *E’* value over the temperature range was observed for composites filled with GnPs modified from solution with BPyBr. When the interactions between polymer and filler cause the restriction of the rubber chains’ mobility, the constrained polymer close to the filler’s surface is thus formed. This may reveal that much stronger interfacial interactions are present in the abovementioned polymer material [32].

## 3. Materials and Methods

### 3.1. Materials

SBR (KER 1500) was supplied by Synthos S.A., Oswiecim, Poland. Sulfur was supplied by Siarkopol, Tarnobrzeg, Poland. Both 2,2′-dibenzothiazyl disulfide (MBTS) and 1,3-diphenylguanidine (DPG) were provided by Akrochem Co., Akron, OH, USA. Graphene nanoplatelets powder (GnPs) in a high-purity form was purchased from XG Sciences Inc., Lansing, MI, USA. 1-butylpyridinium bromide (BPyBr) and 4-methyl-1-butylpyridinium bromide (BmPyBr) were supplied by IoLiTec Ionic Liquids Technologies GmbH, Heilbronn, Germany. Pure acetone (99.5%) was obtained from POCH S.A., Gliwice, Poland.

### 3.2. Fabrication of Graphene Nanoplatelets Modified with BPyBr and BmPyBr from Solution (GnPs/BPyBr and GnPs/BmPyBr, Respectively)

Modified GnPs were obtained from a solution. A quantity of 30 g of GnPs powder and 7.5 g of BPyBr or 8 g of BmPyBr were dispersed in 120 mL of acetone. The protocol of the modification in solution was as follows: the obtained mixture was agitated in an ultrasonic bath (Bandelin Sonorex DT 255 H, Berlin, Germany) twice for 15 min (frequency 35 kHz, 640 W) with a 15 min break. Then, the solution was left to cool down at room temperature. After 48 and 72 h, the dispersion was sonicated again for 15 min. Afterwards, the solvent was evaporated. Finally, the obtained product was dried at 50 °C using a vacuum oven to obtain modified graphene nanoplatelets. All details concerning the method of the filler’s modification and calculation of the amount of BPyBr and BmPyBr deposited onto the GnPs surface were described as well in our previous paper [10].

### 3.3. Preparation of Rubber Mixes and Vulcanizates

The formulas of the filled rubber compounds and the reference sample were as follows: SBR 100 phr; sulfur 2 phr; MBTS 0.5 phr; DPG 0.5 phr; GnPs 0–5 phr; BPyBr 0–1.08 phr; BmPyBr 0–1.15 phr; GnPs/BPyBr 0–6.43 phr; GnPs/BmPyBr 0–5.81 phr. The concentration of GnPs within the modified filler was in the range of 1–5 by weight.

SBR was compounded with the above ingredients using a laboratory two-roll mill (Bridge, UK) at room temperature. The cylinder’s diameter and length were 150 mm and 300 mm, respectively. To avoid scorching and provide effective mixing of all compounds, the rolls were cooled down by circulating water. The friction between the cylinders was 1:1. At the beginning, the raw SBR was masticated, and then, the ingredients were added and mixed continuously. Next, the uncured SBR blends were sheeted out to a thickness of approx. 6–8 mm and left for 24 h at a temperature of 2–6 °C and then heat-cured at 160 °C for the optimum cure time (which was established using rheometric measurements at 160 °C when the samples developed a 90% increase in torque) using an electrically heated hydraulic press [10].

### 3.4. Methods of Characterization

The isotherms of N_2_ adsorption were measured using an ASAP 2420 instrument (Micrometrics, Norcross, GA, USA) at the temperature of −196 °C. These data were used to calculate the micro- and mesoporosity and total pore volume as well. To measure the specific surface area (*S_BET_*) of the fillers, dried powders were degassed at 25 °C under vacuum to remove adsorbed gases. Other textural properties were determined using the standard Barrett–Joyner–Halenda and Dubinin–Astakhov methods.

The temperature and enthalpy of vulcanization were studied using a DSC1 analyzer (Mettler Toledo, Greifensee, Switzerland) equipped with STARe software (Version 10, 2010, Switzerland) by cooling the rubber mixtures to −100 °C at a rate of 10 °C min^−1^ and then heating up to 250 °C at the same rate under nitrogen purging. The weight of each sample was in the range of 7.00–8.35 mg. The samples were placed into aluminum, hermetically sealed calorimetric pans with a capacity of 40 μL. The apparatus was calibrated using standard patterns: indium and n-octane. The onset temperature of the peak referring to the curing reactions was established according to the procedure given in the ISO 11357–1 standard [33]. Thermal parameters of the investigated samples were used to calculate the heat capacity weight fraction of the immobilized rubber layer (*χ)* according to the literature [18].

The thermal stability of vulcanizates and ionic liquids was studied using a TGA/DSC instrument (Mettler Toledo, Switzerland) calibrated with indium and zinc. Samples placed in the open alumina crucibles with a capacity of 70 μL were heated from 25 to 600 °C in an argon atmosphere and then in an air atmosphere from 600 °C to 900 °C with a heating rate of 20 °C min^−1^ (vulcanizates) or 10 °C min^−1^ (ILs) and with a steady gas flow of 50 mL min^−1^.

Dynamic mechanical properties of the investigated composites were observed by a DMA/SDTA 861e analyzer (Mettler Toledo, Switzerland). The tests were carried out using tension deformation mode. Tests were performed using a frequency of 1 Hz and a heating rate of 3 °C min^−1^ from −100 to 70 °C.

## 4. Conclusions

In summary, the textural characteristics and pore size distribution determined from N_2_ adsorption isotherms of pristine and treated GnPs have been evaluated with respect to various adsorption models. The proposed ionic liquids efficiently modified the fillers’ surface. It was obvious that the settlement of BPyBr from the solution onto the GnPs surface led to the restriction of the filler’s microporosity. This resulted in a reduction in GnPs/BPyBr surface area. However, modification with the use of BmPyBr on the one hand caused the formation of more opened filler structure, and, on the other hand, it did not increase the average pore size.

Using GnPs untreated and modified with BPyBr and BmPyBr, as well, SBR composites were successfully prepared. As a result, SBR composites with different thermal properties were obtained. The TGA studies revealed that GnPs modified with ILs in bulk enhanced the thermal stability of the composites. It was proved that GnPs modified with ILs led to reducing the temperature of vulcanization of SBR-based rubber compounds. For fillers modified from solution, this effect was more pronounced.

This work provides a facile method to modify GnPs by using ionic liquids, which may create an opportunity for the preparation of GnPs-based composites having unique properties.

## Figures and Tables

**Table 1:** Textural properties of the studied fillers (*V_max_*—total adsorbed volume, *S_BET_*—specific surface area by BET, *D_BET_* and *D_BJH_*—average pore size obtained by BET or BJH model, *S_DA_*—microporous surface, *V_DA_*—microporous volume).

	BET Model			BJH Model			Dubinin–Astakhov Model	
Filler	*V_max_* (cm^3^ g^−1^ STP)	*S_BET_* (m^2^ g^−1^)	*D_BET_* (nm)	*S_BJH_* (m^2^ g^−1^)	*V_BJH_* (cm^3^ g^−1^)	*D_BJH_* (nm)	*S_DA_* (m^2^ g^−1^)	*V_DA_* (cm^3^ g^−1^)
pristine GnPs	325	298 ± 1	5.5	118.6	0.40	13.5	263	0.11
GnPs/BPyBr	35	30 ± 0.3	31	45.0	0.24	21.4	n.a.	n.a.
GnPs/BmPyBr	623	767 ± 10	5.0	391.0	0.84	8.5	403	0.33

**Table 2:** Parameters of calorimetric measurements (*χ*—fraction of immobilized polymer layer, *T_g_*—glass transition temperature by DSC, Δ*c_p_*—heat capacity, Δ*H*—enthalpy of vulcanization).

SBR Composites	GnPs Content (phr)	Modifier/Method of Modification		*χ* (-)	*T_g_* (°C)	Δ*c_p_* (J g^−1^ K^−1^)	Δ*H* (J g^−1^)
unfilled	0	–	–	0.00	−51.1 ± 0.9	0.42 ± 0.08	5.96 ± 1.50
1 GnPs	1	–	–	0.01	−50.3 ± 0.9	0.41 ± 0.08	12.7 ± 1.50
1 GnPs + BPyBr		BPyBr	mm	0.01	−51.2 ± 0.9	0.41 ± 0.08	7.28 ± 1.50
1 GnPs/BPyBr		BPyBr	s	0.16	−51.4 ± 0.9	0.35 ± 0.08	6.56 ± 1.50
1 GnPs + BmPyBr		BmPyBr	mm	0.11	−51.2 ± 0.9	0.37 ± 0.08	18.44 ± 1.50
1 GnPs/BmPyBr		BmPyBr	s	0.01	−50.3 ± 0.9	0.41 ± 0.08	10.95 ± 1.50
5 GnPs	5	–	–	0.10	−51.3 ± 0.9	0.36 ± 0.08	24.6 ± 1.50
5 GnPs + BPyBr		BPyBr	mm	0.38	−49.9 ± 0.9	0.25 ± 0.08	9.99 ± 1.50
5 GnPs/BPyBr		BPyBr	s	0.04	−51.2 ± 0.9	0.38 ± 0.08	16.28 ± 1.50
5 GnPs + BmPyBr		BmPyBr	mm	0.43	−50.2 ± 0.9	0.23 ± 0.08	12.71 ± 1.50
5 GnPs/BmPyBr		BmPyBr	s	0.07	−51.8 ± 0.9	0.37 ± 0.08	6.29 ± 1.50

mm—melt mixing, s—from solution.

**Table 3:** Thermal stability parameters of investigated SBR composites (*T*_02_, initial decomposition temperature; *T*_50_, 50% decomposition temperature).

SBR Composites	GnPs Content (phr)	Modifier/Method of Modification		*T*_02_ (°C)	*T*_50_ (°C)
unfilled	0	–	–	245 ± 1	441 ± 1
1 GnPs	1	–	–	259 ± 1	445 ± 1
1 GnPs + BPyBr		BPyBr	mm	251 ± 1	441 ± 1
1 GnPs/BPyBr		BPyBr	s	245 ± 1	441 ± 1
1 GnPs + BmPyBr		BmPyBr	mm	235 ± 1	443 ± 1
1 GnPs/BmPyBr		BmPyBr	s	231 ± 1	439 ± 1
5 GnPs	5	–	–	257 ± 1	445 ± 1
5 GnPs + BPyBr		BPyBr	mm	233 ± 1	439 ± 1
5 GnPs/BPyBr		BPyBr	s	229 ± 1	443 ± 1
5 GnPs + BmPyBr		BmPyBr	mm	253 ± 1	443 ± 1
5 GnPs/BmPyBr		BmPyBr	s	237 ± 1	443 ± 1

mm-modification by melt mixing, s-modification from solution.

**Table 4:** Thermal stability parameters of 1-butylpyridinium bromide (BPyBr) and 4-methyl-1-butylpyridinium bromide (BmPyBr) (*T*_02_, initial decomposition temperature, *T_MAX_*, temperature of maximal rate of degradation process).

Ionic Liquid	*T*_02_ (°C)	*T_MAX_* (°C)
BPyBr	213 ± 1	268 ± 1
BmPyBr	153 ± 1	278 ± 1

**Table 5:** DMA-related parameters for investigated SBR composites (*T_g_*, glass temperature transition by DMA, *tan δ_Tg_*, *tan δ* peak height during glass transition, *tan δ_0_*, *tan δ_60_*, mechanical loss factor at 0 °C and 60 °C; standard deviations: *T_g_* ± 1 °C, *tan δ_Tg_* ± 0.09, *tan δ* ± 0.002, *tan δ_60_* ± 0.004).

SBR Composites	GnPs Content (phr)	Modifier/Method of Modification		*T_g_* (°C)	*Tan δ_Tg_* (-)	*Tan δ*_0_ (-)	*Tan δ_60_* (-)
unfilled	0	–	–	−48.4	2.04	0.115	0.099
1 GnPs	1	–	–	−49.4	2.03	0.118	0.118
1 GnPs + BPyBr		BPyBr	mm	−50.3	1.94	0.116	0.065
1 GnPs/BPyBr		BPyBr	s	−49.3	2.03	0.086	0.059
1 GnPs + BmPyBr		BmPyBr	mm	−49.3	1.92	0.093	0.087
1 GnPs/BmPyBr		BmPyBr	s	−46.6	1.96	0.011	0.056
5 GnPs	5	–	–	−48.6	1.88	0.124	0.088
5 GnPs + BPyBr		BPyBr	mm	−50.3	1.97	0.061	0.095
5 GnPs/BPyBr		BPyBr	s	−45.1	1.78	0.112	0.096
5 GnPs + BmPyBr		BmPyBr	mm	−49.2	1.80	0.071	0.059
5 GnPs/BmPyBr		BmPyBr	s	−47.7	1.91	0.106	0.090

mm-modification by melt mixing, s-modification from solution.

## Data Availability

Data are contained within the article.
